# Supplementary material for: Spatiotemporal and kinetic characteristics during maximal sprint running in fast running soccer players
Source: PLoS One. 2025 May 2;20(5):e0322216. doi: 10.1371/journal.pone.0322216 (PMC12047830; doi:10.1371/journal.pone.0322216)
Supplement: S1 Fig — Data points are represented as sprinters (white-filled triangles), fast-running soccer players (black-filled circles), medium-running soccer players (gray-filled circles), and slow-running soccer players (white-filled circles). (DOCX) [file pone.0322216.s001.docx]

**Supplemental data 1.**

Against the percentage of theoretical maximal running speed (%MS_0_), fast-running soccer players exhibited a higher step frequency and a lower step length compared to sprinters. Flight time was shorter in fast-running soccer players than in sprinters, while ground contact time, primarily due to an increased braking time, was longer beyond approximately 90%MS_0_. Regardless of group, net antero-posterior impulse decreased linearly with increasing %MS_0_. On the other hand, vertical impulse lower in fast-running soccer players and sprinters compared to slow-running soccer players. Stance-averaged vertical force was lower in soccer players compared to sprinters, primarily due to reduced vertical force during the propulsive phase.





**Supplemental data 1.** Associations of spatiotemporal variables and ground reaction forces with the percentage of running speed relative to theoretical maximal running speed. Data points are represented as sprinters (white-filled triangles), fast-running soccer players (black-filled circles), medium-running soccer players (gray-filled circles), and slow-running soccer players (white-filled circles).
