# Supplementary material for: Spatiotemporal and kinetic characteristics during maximal sprint running in fast running soccer players
Source: PLoS One. 2025 May 2;20(5):e0322216. doi: 10.1371/journal.pone.0322216 (PMC12047830; doi:10.1371/journal.pone.0322216)
Supplement: Attachment — Submitted filename: ResponseToReviewer_PLoS_rev#1_v2.docx [file pone.0322216.s004.docx]

**Response to the reviewer #1 comments**

We appreciate your thoughtful comments, which have significantly contributed to improving the quality of this paper. First, a reviewer requested group comparisons based on absolute speed values. In response, we added the results of comparisons at 7.5 m/s and 8.0 m/s among soccer players, and at 7.5 m/s, 8.0 m/s, 8.5 m/s, and 9.0 m/s between high-speed soccer players and sprinters. Accordingly, we also expanded the discussion on the sprint mechanics of high-speed soccer players based on absolute speed values. Details are provided below.

**Comment #1**

Although %V0 in the 2nd acceleration phase was similar among the groups, the absolute running velocity was significantly different among the groups. Such differences in the running velocity made the group comparison difficult to interpret. I suggest comparing the groups on a phase of equal absolute velocity.

**Response to comment #1**

In accordance with the reviewer’s comment, we have added data on the absolute values of running speed to the Results section. In the revised text, we examined differences in the measured variables at 7.5 m/s and 8.0 m/s for comparisons among soccer players and at 7.5 m/s, 8.0 m/s, 8.5 m/s, and 9.0 m/s for comparisons between fast-running soccer players and sprinters. Additionally, Figure 2 has been included as the summary of the results.

**Results section:**

**“Comparison between fast-running soccer players and sprinters at the same running speed (Table 3)**

Regardless of running speed, *%MS_0_* was higher in *SOC_High_* compared to *Sp*. No significant differences in ground contact time and flight time were observed between the two groups at any running speed. At 9.0 m/s, braking time was longer but propulsive time tended to be shorter (*p* = 0.062) in *SOC_High_* than in *Sp*. There were no significant differences in step frequency or step length between *SOC_High_* and *Sp* at any running speed, except for 7.5 m/s.

At 9.0 m/s, net antero-posterior impulse, stance-averaged antero-posterior and braking forces were lower in *SOC_High_* compared to *Sp*, but no significant differences were found between the two groups in those at other running speeds. Propulsive impulses at 8.0 m/s and more were lower, and propulsive forces at 7.5 m/s and over were lower in *SOC_High_* than in *Sp*. No significant differences were observed between the two groups in braking impulse at any running speed.

Compared to *Sp*, *SOC_High_* exhibited larger vertical impulse during the braking phase but lower vertical impulse during the propulsive phase at 9.0 m/s, while the corresponding difference was found in vertical impulse on ground contact. There were no significant differences between the two groups in vertical impulse during the braking or propulsive phases at any running speed. At 9.0 m/s, the averaged vertical force during the propulsive phase was lower in *SOC_High_* compared to *Sp*, while no significant differences were found between the two groups in the stance-averaged vertical and vertical forces during the braking phase.**”** (Line. 262-280)

**“****Comparison between fast- and slow-running soccer players at the same running speed (Table 4)**

Regardless of running speed, *%MS_0_* was lower in *SOC_High_* compared to *SOC_Low_*. No significant differences were observed between the two groups in ground contact time and flight time. Braking time was shorter in *SOC_High_* compared to *SOC_Low_*, while propulsive time was longer. *SOC_High_* exhibited higher step frequency compared to *SOC_Low_*. Step length at 8.0 m/s tended to be shorter in *SOC_High_* than in *SOC_Low_*, but the difference was not significant (*p* = 0.070).

At 7.5 m/s and 8.0 m/s, net antero-posterior impulse was greater in *SOC_High_* compared to *SOC_Low_*, primarily due to lower braking impulse and larger propulsive impulse in the former. The stance-averaged antero-posterior forces at the two running speeds were higher in *SOC_High_* than in *SOC_Low_*. At 8.0 m/s, propulsive force was higher in *SOC_High_* than in *SOC_Low_*, and braking force tended to be lower in the former compared to the latter (*p* = 0.060).

At 7.5 m/s and 8.0 m/s, vertical impulses during the braking phase were lower and those during the propulsive phase were higher in *SOC_High_* than in *SOC_Low_*. There was no significant difference in stance-averaged vertical force between the two groups. However, the averaged vertical force during the braking phase was smaller and that during propulsive phase was greater in *SOC_High_* compared to *SOC_Low_*.**”** (Line. 284-300)

With changing this revision, we have added details on the analysis methods in the Methods section and included a discussion on group comparisons at the same running speed in the Discussion section.

**Methods section:**

To examine group differences in each variable at the same running speed, we interpolated each variable as a function of running speed within the data range of each participant. From the interpolated data, we extracted the measured variables at 7.5 m/s, 8.0 m/s, 8.5 m/s, and 9.0 m/s. In *SOC_Low_*, the running speed of two participants did not reach a maximum of 8.0 m/s. In *SOC_Med_*, three participants did not reach 8.5 m/s, while in *SOC_High_*, two participants did not reach 9.0 m/s. In *Sp*, one participant also did not reach 9.0 m/s. Therefore, the independent variables at the corresponding running speed were treated as missing values. To determine the theoretical maximal running speed (*MS*_0_), the stance-averaged horizontal forces were plotted against running speed and fitted linearly with a first-order polynomial function. The y-intercept was computed as *MS*_0_. The running speed in each phase was also expressed as the value relative to *MS*_0_ (%*MS*_0_).

(Line. 152-161)

**Discussion section:**

“**Factors contributing to differences in maximal running speed between fast-running soccer players and sprinters**

In this study, we compared spatiotemporal variables and ground reaction forces between *SOC_High_* and *Sp* with relation to the same running speed and the same running phase. In this section, we discuss the factors contributing to the lower maximal running speed of *SOC_High_* compared to *Sp*, based on the findings of this study. As seen in Fig. 1, *SOC_High_* exhibited a higher step frequency than *Sp* up to 7.5 m/s; however, beyond this speed, no significant difference was observed. Additionally, in *SOC_High_*, step frequency decreased from *A_2nd_* to *MS*, whereas it remained constant during the corresponding phase in *Sp*. This decrease may be attributable to a longer ground contact time in *SOC_High_*, likely due to lower vertical forces, particularly during the propulsive phase. As greater vertical force during ground contact was associated with a shorter ground contact time above mentioned, it is assumed that *SOC_High_* may be unable to reduce their ground contact time due to lower vertical force during the propulsive phase, which may have prevented them from maintaining step frequency.

Another mechanism underlying the difference in maximal running speed between *SOC_High_* and *Sp* may be the difference in net antero-posterior impulse at the same running speed. At 9.0 m/s, *SOC_High_* exhibited a lower net antero-posterior impulse, primarily due to a lower propulsive impulse compared to *Sp*. Given these findings, the lower maximal running speed in *SOC_High_* may be attributable to the inability to generate sufficient acceleration to achieve higher speeds beyond *A_2nd_*. Taken together, fast-running soccer players exhibit lower acceleration capability beyond the 2^nd^ acceleration phase and reduced vertical force during the propulsive phase, which may contribute to their lower maximal running speed compared to sprinters.” (Line 422-443)

“**Factors contributing to differences in maximal running speed between fast- and slow-running soccer players**

In this section, we discuss the factors contributing to the lower maximal running speed of *SOC_High_* compared to *SOC_Low_*, based on the findings of this study. As shown in Fig. 1, *SOC_High_* exhibited a higher step frequency during entire sprint running. This may be due to a shorter flight time in *SOC_High_*, primarily resulting from lower vertical impulse, as no significant differences were observed in ground contact time between the two groups. Additionally, beyond *A_2nd_*, *SOC_High_* exhibited higher vertical forces during ground contact, particularly in the propulsive phase, leading to a shorter ground contact time compared to *SOC_Low_*. Therefore, the higher step frequency in *SOC_High_* beyond the 2^nd^ acceleration phase can be attributed to greater vertical force.

In both groups, step length increased with running speed, and *SOC_High_* was able to extend their step length even further as running speed increased. Both antero-posterior and vertical impulses are determinants of step length [22]. Since vertical impulse was lower in *SOC_High_* compared to *SOC_Low_*, the longer step length in *SOC_High_* may be attributed to a greater net antero-posterior impulse. In particular, *SOC_High_* exhibited less braking impulse due to brief braking time and greater propulsive impulse resulting from both longer propulsive time and propulsive force up to the *A_2nd_*. Based on these findings, fast-running soccer players can produce greater propulsive force throughout the entire running phase and generate larger vertical forces at higher speeds compared to slow-running soccer players.” (Line 445-463)

”

**Comment #2**

L37. The vertical ground force was “smaller” in SOChigh than in sprinters, not “reduced”.

**Response to comment #2**

We have modified.

**Comment #3**

L38-40. I couldn’t understand this conclusion. In the present results, the vertical ground force during the maximal speed phase was significantly smaller in SOChigh than in sprinters (Table 2).

**Response to comment #3**

As mentioned above, we have added group comparisons at the same running speed. Therefore, we have revised this part as follows:

“During the initial acceleration phase, *SOC_High_* exhibited similar running speeds to *Sp*. This was attributed to a higher step frequency in *SOC_High_* compared to *Sp*, while net antero-posterior impulse was lower in the former than in the later. In the range of running speed from 7.5 m/s to 8.5 m/s, net antero-posterior impulse for *SOC_High_* was similar to that for *Sp*. At 9.0 m/s, *SOC_High_* exhibited a lower net antero-posterior impulse compared to *Sp*, primarily due to a reduced propulsive impulse. Additionally, vertical impulse during the braking phase was larger in *SOC_High_* compared to *Sp*, due to a longer braking time, while vertical impulse during the propulsive phase was smaller, due to a tendency for a reduced propulsive time and vertical force during the corresponding phase. Compared to *SOC_Low_*, *SOC_High_* exhibited higher step frequency through sprint running and longer step lengths from the 2nd acceleration phase to maximal speed phase. Additionally, net antero-posterior impulse at the same running speed was greater in *SOC_High_* compared to *SOC_Low_*. Vertical impulse was lower during the braking phase but higher during the propulsive phase in *SOC_High_* than in *SOC_Low_*. Thus, the sprint mechanics of *SOC_High_* is characterized by a similar ability of speed acquisition up to the 2^nd^ acceleration as sprinters. However, at 9.0 m/s or over, *SOC_High_* exhibits a greater vertical impulse, leading to a lower step frequency.”

(Line 30-45)

**Comment #4**

L54. Although the authors defined three primary phases, the text after this point described only two phases (acceleration and maximal speed phases).

**Response to comment #4**

We have rewritten the corresponding part as follows:

“For example, it has been reported that the antero-posterior ground reaction forces (GRFs) varied between faster and slower sprinters throughout the acceleration phase [18]. Further, the propulsive and braking forces were lower in soccer players than in sprinters at equivalent speed (8 and 8.5 m/s) during the 2^nd^ acceleration phase [15]. During the maximal speed phase, non-sprinters exhibit longer contact times and shorter step lengths compared with sprinters [19]. As compared to non-sprinters, sprinters demonstrate larger vertical GRFs and exert greater vertical GRFs during the first half of ground contact in the maximal speed phase [16]. Furthermore, elite soccer players demonstrate a higher theoretical maximal horizontal force derived from horizontal force-velocity relationship compared with sub-elite soccer players [17], indicating that the formers have greater acceleration capability in the initial acceleration phase.” (Line 69-78)

**Comment #5**

L68-72. Please provide the phases (the 1st acceleration, the 2nd acceleration or the maximal speed phases) of these findings.

**Response to comment #5**

Please refer to the response to comment #4.

**Comment #6**

L73-75. Please provide appropriate references for this statement.

L75-76. Please add appropriate reference(s) for this statement.

**Response to comment #6**

We have done.

**Comment #7**

L96. Is “The participants” the sprinters?

**Response to comment #7**

We have revised the following sentence.

“All participants underwent their specific training 5-6 times per week, with each session lasting 1.5-2 hours.” (Line 99-100)

**Comment #8**

L116-117. How do the authors think about the effects of different types of shoes on the present findings?

**Response to comment #8**

Regarding this point, we acknowledge that difference in footwear might influence ground reaction forces. However, we could not find any previous studies concerning the effect of different shoes on ground reaction forces. Hence, while we cannot definitively determine whether a difference exists, we have added the following sentences to the Limitation section.

“Second, the difference in ground reaction forces between sprinters and soccer players might be influenced by footwear (spiked shoes vs. running shoes). To the best of our knowledge, no study has explicitly examined this factor. In this study, participants were instructed to wear their regularly used shoes to ensure familiarity and natural sprint performance.”

(Line 471-474)

**Comment #9**

L133-134. How much did the center of pressure move in a single step? If the position of the center of pressure moved from the time of ground contact to the takeoff, it should affect the step length determined by the method described here.

**Response to comment #9**

Regarding the reviewer’s comment, the center of pressure (COP) shifted by approximately 0.1–0.2 m during ground contact, regardless of running speed. In this study, we analyzed the midpoint between touchdown and toe-off for all steps. The effect of COP shift is consistently present in all steps. In this part, we have rewritten the following sentence in the *Methods* section.

“step length was determined as the distance between the midpoint of ground contact and takeoff in one step and the corresponding midpoint in the next step.”

(Line 141-142)

**Comment #10**

P141-142. “speed” and “velocity” are mixed in this manuscript. The typical example is “maximal speed (Vmax)”. However, these terms are distinctly different. “speed” is a scalar and “velocity” is a vector.

**Response to comment #10**

In this study, we have standardized the term ‘maximal speed’. Additionally, we used the abbreviation ‘MS’ to represent the maximal speed phase.

**Comment #11**

L146-147. Is this correct? For example, is the slope F0?

**Response to comment #11**

According to the reviewer’s comment #18, this section was removed.

**Comment #12**

L147. Please include appropriate reference(s) for the Pmax calculation.

**Response to comment #12**

According to the reviewer’s comment #18, this section was removed.

**Comment #13**

L160. What is “time” in this context? Phase?

**Response to comment #13**

There was an error in the expression. The correct terms are 'group’ and ‘phase’. We have corrected in the revised text.

**Comment #14**

L168. “IBM”

**Response to comment #14**

We sincerely appreciate you pointing out the incorrect notation. We have done.

**Comment #15**

Results. Showing the ground reaction force profiles during a single step for each phase may help the readers understand the results.

**Response to comment #15**

We have added representative data on antero-posterior and vertical ground reaction forces for a sprinter, a fast-running soccer player, and a slow-running soccer player across the analyzed steps to the revised text.

**Comment #16**

Figure 1. Please provide a higher-resolution image.

**Response to comment #16**

We have done.

**Comment #17**

L208-209. What is “the stance-averaged antero-posterior force net antero-posterior impulse”?

**Response to comment #17**

There was an error in the expression. We have modified the following sentence.

“There were no significant differences between *SOC_High_* and *Sp* and between *SOC_High_* and *SOC_Low_* in each of the net antero-posterior impulse, braking impulse and propulsive impulse (Table 2).” (Line 233-234)

**Comment #18**

L235-241. There was no discussion regarding the horizontal force-velocity profile. The authors should reconsider the need for this data.

**Response to comment #18**

As the reviewer pointed out, we did not discuss the horizontal force-velocity profile in the text. Since we have added group comparisons of the measured variables at the same speed, we have considered that presenting the results related to the horizontal force-velocity profile was unnecessary. Therefore, we removed the descriptions of the horizontal force-velocity profile from the revised text. However, since we calculated the estimated maximal running speed and used it to normalize running speed, we have retained the description of this calculation method.

**Comment #19**

L257-262. I think the group difference in step frequency was simply due to the difference in contact time. There is no need to discussion of vertical impulse.

**Response to comment #19**

We have agreed and deleted the description in the revised text.

**Comment #20**

L270-271. The effect size is only reported in limited places in the manuscript. Please provide the effect size for the other comparisons.

**Response to comment #20**

We determined that presenting effect size for all results would be complex. Therefore, we initially reported effect size only for results showing a significant tendency. However, in the revised text, we have decided that it would be more appropriate to base our discussion on statistical significance. As a result, we excluded the discussion based on effect sizes.

**Comment #21**

L272-276. Again, I think the group difference in step frequency was simply due to the difference in contact time.

**Response to comment #21**

We have agreed and deleted the description in the revised text.

**Comment #22**

L287-289. How about sprint running on the ground, not the treadmill?

**Response to comment #22**

We have considered that the relationship between stance-averaged vertical force and ground contact time is consistent not only on a treadmill but also in overground sprinting. In fact, our data showed that in the 2^nd^ acceleration phase, a higher stance-averaged vertical force was associated with shorter ground contact time (*r* = 0.726 for soccer players). Since the previous study cited here was conducted using a treadmill and during the maximal speed phase, we have included an explanation to demonstrate how this relationship is reflected in our data as well.

“*SOC_High_* had shorter ground contact time, and higher stance-averaged vertical force as compared to *SOC_Low_*. The stance-averaged vertical force was negatively related to ground contact time in the *A_2nd_* (r = 0.726 for soccer players) (unpresented data). These indicate that as compared to *SOC_Low_*, *SOC_High_* can produce greater vertical force within a short ground contact time.” (Line 348-352)

**Comment #23**

L293-296. Please specify the phase of the sprint running regarding the previous findings.

**Response to comment #23**

We have changed this part to the following sentence.

“Net antero-posterior impulse is associated with the subsequent change in running speed during the entire acceleration phase [23].” (Line 355-356)

**Comment #24**

L300-304. The impulse is equal to the change in momentum (mass times velocity). But the running velocity itself was different among the groups in the 2nd acceleration phase. Hence, it is difficult to discuss the group difference from the viewpoint of impulse (equal to changes in velocity). I suggest to compare the groups on a phase of equal absolute velocity (e.g., 7.5-7.9 m/s), not on a phase of equal relative velocity (%V0). At 7.9 m/s of running velocity, SOClow cannot accelerate at all, but SOChigh can still accelerate. The difference and its mechanism are scientifically interesting.

**Response to comment #24**

We have agreed and added the results of group differences in the measured variables at the same running speeds in the revised text. Please refer to the response to the comment #1.

**Comment #25**

L304. “shorter” step length rather than “lower”

**Response to comment #25**

We have revised.

**Comment #26**

L318-321. Again, I suggest to directly (statistically) compare the groups on a phase of equal absolute velocity (e.g., 8.5-8.8 m/s), as the authors stated.

**Response to comment #26**

We have agreed and added the results of group differences in the measured variables at the same running speeds in the revised text. Please refer to the response to the comment #1.

**Comment #27**

L347-349. Are these previous findings obtained from sprint running?

**Response to comment #27**

Yes, the previous study focused on sprint running.

**Comment #28**

L354-356. Sprinters exhibited greater vertical force not only in the braking phase but also in the propulsive phase.

**Response to comment #28**

As the reviewer pointed out, sprinters exhibited lower vertical force than fast-running soccer players in both the braking and propulsive phases. Therefore, we have revised the text as follows:

“The vertical force during the braking phase tended to be lower in *SOC_High_* than in *Sp*. Clark and Weyand [16] demonstrated a positive correlation between maximal running speed and stance-averaged vertical force during the braking phase. Additionally, *SOC_High_* exhibited a lower vertical force during the propulsive phase compared to *Sp*. The development of maximal speed during sprinting is accompanied by an increase in vertical stiffness [30]. Based on these findings, it is likely that fast-running soccer players cannot stiffen their lower limbs particularly during the maximal speed phase, and consequently generate lower vertical forces, and experience longer ground contact times compared to sprinters.”

(Line 413-420)

With changing it, we have added the following reference to the revised text.

**Comment #29**

L356-358. I think that the vertical force and reactive strength are not independent with each other. The greater vertical force during sprint running may result from the greater reactive strength.

**Response to comment #29**

We have agreed. As the reviewer pointed out, the two variables are likely not independent of each other. One possible reason why soccer players exhibit lower vertical force than sprinters during sprinting is their lower eccentric muscle strength. Therefore, we have revised the discussion in this part. Please refer to the response to the comment #28.

**Comment #30**

L359. What is “requisite reactive strength”?

**Response to comment #30**

This word was removed during the revision process.

**Comment #31**

L370-373. The lack of significant difference is NEVER the study limitation. The lack of discussion about the reported data is a serious flow of the manuscript.

**Response to comment #31**

This part was removed during the revision process. We have revised as follows:

“There are some limitations in this study. First, we did not have data on sprint running kinematics. A study by Garden [31] has shown that the kinematics of sprint running may differ between sprinters and soccer players, which could explain the differences in spatiotemporal and mechanical variables observed in this study. In particular, biomechanical analysis is needed to investigate the factors contributing to the increase in braking time and the reduced propulsive time from the 2^nd^ acceleration phase to the maximal speed phase. Second, the difference in ground reaction forces between sprinters and soccer players might be influenced by footwear (spiked shoes vs. running shoes). To the best of our knowledge, no study has explicitly examined this factor. In this study, participants were instructed to wear their regularly used shoes to ensure familiarity and natural sprint performance. Finally, this study examined regional collegiate soccer players, whereas the previous study included regional to national level players [17]. Further research is needed to clarify these points.”

(Line 466-476)

**Comment #32**

L381-382. From which data the authors state so? “6 m/s”?

**Response to comment #32**

This speed was shown through visual inspection of the graph. As the response to the comment#18 was stated, the horizontal force-velocity profile has been excluded in the revised text. Therefore, we have also revised the description in this section.

**Comment #33**

L382-384. The authors did not state so in the discussion. Not only SOChigh, but all the groups showed decreases in the step frequency.

**Response to comment #33**

As the reviewer pointed out, the *Perspective* section was constructed with content that had not been explained in the discussion. Since we determined that the content in this section extended beyond the results of this study, we decided to remove it. Instead, we have included specific points for future investigation in the limitations section. Please refer to the response to comment #31

**Comment #34**

L397. No significant difference was found in the step frequency during the 2nd acceleration phase.

**Response to comment #34**

We have revised the *Conclusion* section as follows:

“Sprint mechanics of fast-running soccer players is characterized by similar ability of speed acquisition up to the 2^nd^ acceleration to sprinters but, at above 9.0 m/s and more, they exhibit an increase in vertical impulse during ground contact, leading to a lower step frequency. Additionally, fast-running soccer players produce greater acceleration capacity, lower vertical force during the braking phase, and higher vertical force during the propulsive phase compared to slow-running soccer players.” (Line 479-484)

**Response to the reviewer #2 comments**

We appreciate your thoughtful comments, which have significantly contributed to improving the quality of this paper. First, another reviewer requested intergroup comparisons based on absolute speed values. In response, we added the results of comparisons at 7.5 m/s and 8.0 m/s among soccer players, and at 7.5 m/s, 8.0 m/s, 8.5 m/s, and 9.0 m/s between high-speed soccer players and sprinters. Accordingly, we also expanded the discussion on the sprint mechanics of high-speed soccer players based on absolute speed values. Details are provided below.

**Comment #1**

A limitation is that the soccer players wore running shoes and the sprinters spikes, but I anticipate this will be discussed. Moreover, many of the more subtle differences found among the groups probably are dependent upon these specific groups of soccer players and sprinters. I noticed that this point wasn’t addressed at all, I think it should be discussed.

**Response to comment #1**

We agree with this comment and have added a note in the limitations section regarding the potential influence of spiked shoes, although we could not find any previous studies concerning the effect of different shoes on ground reaction forces.

“Second, the difference in ground reaction forces between sprinters and soccer players might be influenced by footwear (spiked shoes vs. running shoes). To the best of our knowledge, no study has explicitly examined this factor. In this study, participants were instructed to wear their regularly used shoes to ensure familiarity and natural sprint performance.”

(Line 471-474)

**Comment #2**

Although the methodology has been used and described in previous work, I still think that the manuscript would be improved with inclusion of a figure showing ground reaction forces and some explanation of the different parameters investigated.

**Response to comment #2**

We have added representative data on antero-posterior and vertical ground reaction forces for a sprinter, a fast-running soccer player, and a slow-running soccer player across the analyzed steps to the revised text.

**Comment #3**

I do not think that table 3 adds very much (I am aware that this force profiling is frequently used but in a way it just summarizes differences in acceleration and maximal speed capacity among athletes. It involves extrapolation and the assumption of linearity. I am curious to read how the authors are going to explain differences and/ or lack thereof among groups. (lines 367-375), I am slightly disappointed, why not pointing out that this method has inherent uncertainties? Why not just leave Table 3 and these results out of the study, which would also give room for a figure (point 2)

**Response to comment #3**

We added an analysis at the same running speed in response to another reviewer’s comment. As a result, Table 3 and the descriptions related to the horizontal force-velocity relationship were removed in the revised text.

In the revised text, we added results and discussion on the group-related differences in spatiotemporal variables and ground reaction forces at the same running speed.

**Comment #4**

Perspective:’ Therefore, conducting assisted sprinting and plyometric training that focuses on the vertical component of force development might benefit for fast-running soccer players in improving maximal running speed.’. I think the authors should address that maximal sprint speed is of far less importance for soccer players than acceleration, it may not be warranted to invest much specific training to increase Vmax for soccer players.

**Response to comment #4**

As another reviewer pointed out, the *Perspective* section was constructed with content that had not been explained in the discussion. Since we determined that the content in this section extended beyond the results of this study, we decided to remove it. Instead, we have included specific points for future investigation in the limitations section.

“There are some limitations in this study. First, we did not have data on sprint running kinematics. A study by Garden [31] has shown that the kinematics of sprint running may differ between sprinters and soccer players, which could explain the differences in spatiotemporal and mechanical variables observed in this study. In particular, biomechanical analysis is needed to investigate the factors contributing to the increase in braking time and the reduced propulsive time from the 2^nd^ acceleration phase to the maximal speed phase. Second, the difference in ground reaction forces between sprinters and soccer players might be influenced by footwear (spiked shoes vs. running shoes). To the best of our knowledge, no study has explicitly examined this factor. In this study, participants were instructed to wear their regularly used shoes to ensure familiarity and natural sprint performance. Finally, this study examined regional collegiate soccer players, whereas the previous study included regional to national level players [17]. Further research is needed to clarify these points.”

(Line 466-476)

**Comment #5**

Minor. Typo error in the following, surely fast running soccer player produce vertical forces in the 2nd acceleration and the maximal speed phases, fast-running soccer players would not produce vertical force and would have lower step frequency and step length compared with sprinters.

**Response to comment #5**

This point highlighted a mistake in the description. We have corrected it as follows.

“2) in the 2^nd^ acceleration and the maximal speed phases, fast-running soccer players would produce lower vertical force and would have lower step frequency and step length compared with sprinters.”

(Line 91-93)
